# Cats Have Increased Protein Digestibility as Compared to Dogs and Improve Their Ability to Absorb Protein as Dietary Protein Intake Shifts from Animal to Plant Sources

**DOI:** 10.3390/ani10030541

**Published:** 2020-03-24

**Authors:** Christina Golder, James L. Weemhoff, Dennis E. Jewell

**Affiliations:** 1 Hill’s Pet Nutrition Inc., Topeka, KS 66603, USA; chris_golder@hillspet.com (C.G.); james_weemhoff@hillspet.com (J.L.W.); 2 Department of Grain Science and Industry, Kansas State University, Manhattan, KS 66506, USA

**Keywords:** canine, feline, protein digestibility

## Abstract

**Simple Summary:**

Because dogs are omnivores and cats are obligate carnivores, it is of value to pet owners and nutritionists to know how well they digest protein from plants and animals. This study evaluated the difference in digestibility using plant and animal protein sources, which are used in the pet food industry. These plant and animal sources resulted in protein digestibility that met or exceeded that expected for dogs and cats. As previously shown, cats had superior protein digestibility as compared to dogs. Regarding the difference in digestibility between the proteins from plants or animals—as a class, there was no difference between plant and animal protein in dogs. However, in cats, the protein from plants was more highly digested than animal protein.

**Abstract:**

This retrospective study used 226 dogs and 296 cats to evaluate whether protein absorption was influenced by species, and within species, what influence increasing the percentage of total dietary protein, as plant protein, had on protein absorption. Each food was evaluated by at least one study with a minimum of six dogs or cats assigned to each study. Dietary inclusion of animal and plant based protein was calculated by analysis of ingredients and dietary inclusion level. Both dogs and cats were able to digest dietary plant protein, with protein digestibility in dogs unchanged as plant protein increased, while in cats, eating dry food, an increase in plant protein, was associated with increased protein digestibility. When individual plant high-concentration protein sources (excluding the protein from whole grains) were evaluated (i.e., soybean meal, soybean protein isolate, corn gluten meal, and rice protein concentrate) there was no response to increasing protein from these sources in the dog. In the cat, there was a significant positive effect on protein digestibility associated with an increasing concentration of corn gluten meal. In summary, as the dietary protein shifted from striated muscle and other animal proteins to plant based proteins, there was no effect in the dog, while in cats, increasing dietary plant protein was associated with increasing protein digestibility (5.5% increase at 50% protein from plants in dry cat food). Protein digestibility of food in dogs and cats is similar, if not enhanced, when the plant protein sources are concentrated from soybeans (soybean isolate, soybean meal), corn (corn gluten meal), or rice (rice protein concentrate).

## 1. Introduction

Commercial pet foods are the preferred nutrient sources for many pet owners and there is a great variance in the amount of dietary protein that is provided by plant and animal sources in these foods. Protein adequacy is a function of the amino acid makeup and of protein digestibility; both animal and plant protein sources have been used to meet these needs in dog and cat foods. However, the relative digestibility of these ingredients are not clearly established. The Association of American Feed Control Officials (AAFCO) [1] and the European Pet Food Industry Federation (FEDIAF) [2] have published dietary minimums for adequate amino acids and protein concentrations. However, there is no regulatory standard for the percent of protein, which should come from plants or animals. There is an expectation regarding digestibility, for example, FEDIAF states that an apparent protein digestibility of 80% was used to establish the protein minimum recommendation [2] (page 10). Digestible protein can be expressed as a percent and refers to the amount of protein in the feces in comparison to the amount of protein consumed. True protein digestibility is calculated by subtracting an estimate of the metabolic protein contained in the feces from the measured fecal protein concentration. True protein digestibility has the advantage of predicting the actual protein digestibility of the food and is needed when comparing foods of greatly different protein concentration in order to not artificially inflate the protein digestibility of high protein foods as compared to reduced protein foods. True protein digestibility accounts for fecal metabolic protein, which represents a higher percentage of total fecal protein in low dietary protein foods when compared to foods of higher protein concentration. 

Although protein adequacy requires the correct amino acids to be absorbed in their appropriate concentrations, the total tract protein digestibility is a valuable indicator of protein nutrition. The recommended minimum concentrations have been shown to be more than adequate with normal pet food ingredients in the dog [3] and the cat [4] in foods with protein digestibility similar to the averages reported by Hall et al. [5]. 

This study evaluates the influence of the dietary source of protein, specifically evaluating the influence of animal protein or plant protein on subsequent whole tract protein digestibility with crude fiber also included in the analysis. Because the experimental unit is a group of pets (minimum of *n* = 6) with variable ages, pet age was not a part of this evaluation.

## 2. Materials and Methods

These protocols were approved by the Institutional Animal Care and Use Committee, Hill’s Pet Nutrition, Inc., Topeka, KS, USA (Permit Numbers: CP13, CP14). The dogs used in this research were immunized against *Rabies* (rabies), *Bordetella* (bordetella), *Protoparvovirus* (parvovirus), *Adenoviridae* (adenovirus), and *Canine morbillivirus* (canine distemper). All cats were immunized against *Rabies* (rabies), *Felid alphaherpesvirus 1* (viral rhinotracheitis), *Feline caliciviridae* (feline calicivirus), and *Feline panleukopenia* (feline panleukopena virus). No pets had chronic systemic disease, which was evaluated with annual urinalysis, serum biochemical analyses, complete blood count determination, and physical examination. Cats were housed individually but had access to group socialization, interaction with animal care technicians, and toys. Dogs were housed individually and had access to exercise in groups, interaction with animal care technicians, and toys. Dogs and cats were in facilities with varied seasonal changes through access to natural light. Some of these studies were used in the 2013 evaluation of metabolizable energy predictive equations [5]. 

All studies were conducted over a period of 110 months. The canine studies used 226 healthy dogs and the feline studies used 296 healthy short hair domestic cats (Table 1). The dogs included 20 intact females, 102 spayed females, 11 intact males, and 93 neutered males. The cats included 27 intact females, 137 spayed females, 2 intact males and 130 neutered males. 

There were 459 canine studies and 427 feline studies. The studies used both canned and dry foods with varying nutrient compositions. There were non-commercial (not available for purchase) and commercial pet foods tested. All of the foods tested met the minimum requirements for complete and balanced nutrition for adult dogs or cats established by the Association of American Feed Control Officials. One food met the minimum requirements for both dogs and cats. Pet food chemical composition is reported in Table 2.

All studies used the AAFCO quantitative collection protocol with a minimum of six adult dogs or cats. In this protocol there are two phases. The pre-collection phase is at least seven days and allows the pets to become acclimated to the test food; food intake is adjusted as needed, so weight is neither gained nor lost. The next phase of 5 days (120 h) is used for total fecal collection. Food intake is controlled by not changing the food offering (which is based on amount needed to maintain weight from the earlier phase). Water is always available. The measurements for ash, crude fiber, fat, protein, moisture and energy were completed by commercial laboratories (Eurofins Scientific, Inc., Des Moines, IA, USA or Ontario Nutri Lab, Inc., Fergus, ON, Canada) using official methods of analysis published by AOAC International. True protein digestibility was calculated by subtracting an estimate of the metabolic protein contained in the feces from the measured fecal protein concentration. It is calculated as follows:

True protein digestibility = [(protein intake − (fecal protein − endogenous metabolic protein))/protein intake](1)

True protein digestibility is different than apparent protein digestibility, which does not take into account endogenous metabolic protein. Apparent protein digestibility is calculated as follows:

Apparent protein digestibility = [(protein intake − fecal protein)/protein intake](2)

In order to correct for endogenous metabolic fecal protein (fecal protein not of dietary origin) the endogenous protein correction of 63 mg nitrogen/kg weight to the ¾ power suggested by Kendall et al. was used [6]. This value is in the range of estimates for metabolic fecal protein for the dog and cat [6,7,8].

For the purposes of this study, plant and animal proteins were categorized as follows: animal protein sources include meat, organs, meat meals, meat byproducts and blends of poultry, fish, beef, pork, lamb, and venison. All egg and dairy proteins were also categorized as animal. Plant protein sources include flours, starches, fibers, protein isolates, blends, and whole fruits. Whole grains and vegetables from rice, corn, soy, wheat, pea, potato, flax, algae, apple, sunflower, apple, pecan, tomato, pumpkin, spinach, ginger, rye, citrus, cranberry, sweet potato, green bean, chickpea, bell pepper, quinoa, carrot, zucchini, and sorghum were all categorized as plant sources. Crystalline amino acids were not classified as plant or animal.

Multiple linear regression analysis was performed using stats models 0.9.0 in Python 3.7.3 [9,10,11] to estimate the relationships between protein digestibility and dietary crude fiber, percent of dietary protein from plants, and the potential interaction between crude fiber with percent of dietary protein from plants. The statistical significance of regression coefficients was evaluated by a two-tailed t-test with a significance level of 0.05.

## 3. Results

The true protein digestibility means and standard deviations for the studies are reported in Table 3. Because it accounts for fecal metabolic protein and does not vary in response to dietary protein intake, true protein digestibility was used for evaluation. There was an increased protein digestibility in the feline as compared to the canine (*p* < 0.01) for both wet and dry foods. Of interest, there was one food that was designed to be complete and balanced for both dogs and cats, which was tested in both species. This food had an average protein digestibility of 88% in dogs and 96.1% in cats. This food (Prescription Diet^®^ a/d^®^ from Hill’s Pet Nutriton, Manufactured in Topeka, Kansas, USA, had ash 1.7%, crude fiber 0.1%, fat 7.0%, moisture 78.5%, protein 10.1%, and gross energy 1337.7 kcal/kg).

To evaluate the relationship between dietary protein source and protein digestibility, dietary crude fiber was used with percentage of protein from plants and their interaction in the multivariable analysis. This was done because the addition of plant proteins, especially when coming from whole grains, often brings an increase in dietary fiber. In order to account for this, both the percent of plant protein and percent of food as crude fiber were used along with their interaction as independent factors in the regression analysis. In both dry and wet dog food, there was not a significant relationship between percent of the food as fiber or the percent of the protein from plants with protein digestibility (Table 4).

There was a negative effect of crude fiber on protein digestibility in both dry and wet cat food. There was a positive effect of plant protein (as well as a positive effect of the interaction of fiber and plant protein) in dry cat food on protein digestibility (Table 5). An example of protein digestibility changes with fiber and plant protein in feline dry foods is: if crude fiber was at the mean (and plant protein was not present) the protein digestibility would be at 89.7%, if plant protein was at 50% (and crude fiber remained at the mean concentration) the predicted protein digestibility is 95.2%.

**Figure 1:**
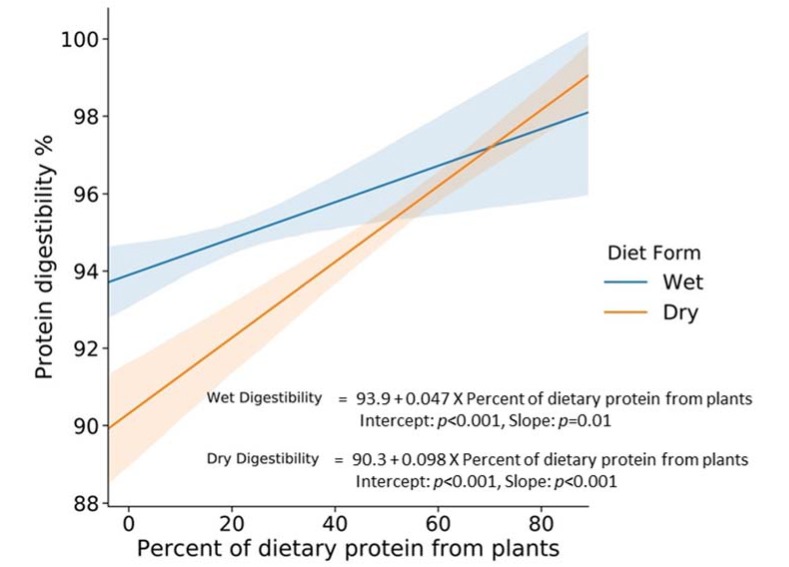
The effect of dietary plant protein on protein digestibility in cats.

To illustrate the overall effect of plant protein on protein digestibility in the cat, the graph of the relationship between protein digestibility and percent of dietary protein from plants is shown in Figure 1. In this analysis, which is the regression analysis of protein digestibility percent, and the percent of dietary protein from plants (no interaction term), the slopes are 0.098, 0.047 with *p* values of *p* < 0.001, *p* = 0.01 for dry and canned foods, respectively.

The further evaluation of the effect of plant protein was accomplished through looking at the influence of individual whole grains or plant protein fractions. The response of protein digestibility in the dog is expressed in Table 6. There was a positive slope associated with the inclusion of protein from dietary rice grain and a negative association with increasing dietary protein from whole corn. If 50% of the protein came from these grains (and other dietary factors unchanged) it would result in an increased protein digestibility of 5.2% for rice and a decrease of 4.2% for corn. The protein fractions (rice protein concentrate, corn gluten meal, soybean meal, and soybean protein isolate) did not have a statistically significant effect on protein digestibility in the dog.

The response of protein digestibility in the cat is expressed in Table 7. In the cat, there was a negative response to increasing protein from whole rice grain and a positive response to protein from corn gluten meal. If these dietary ingredients accounted for 50% of the protein, the predicted protein digestibility for the whole grain rice formula is 87.7% and for the corn gluten meal formula is 96.8%.

## 4. Discussion

In this analysis, protein digestibility is influenced by the source of protein and the species being fed. For example whole grains may have a positive effect as seen with rice in the dog, or a negative effect as seen with corn in the dog, and rice in the cat. However, the proteins themselves, as seen by the evaluation of rice protein concentrate and corn gluten meal, have either no effect or a positive effect on protein digestibility. This suggests that the non-protein fraction of the whole grain is influencing protein digestibility.

When high starch flours (varying in protein concentration from 5.6% to 13.1%) were included in canine diets (from 43.1% to 53.6%) there was a reduction in protein digestibility when potato was used. However, foods formulated with rice, corn, sorghum, barley, and wheat were not different from each other in protein digestibility [12]. The protein digestibility of dog food was not changed with increasing soybean meal concentration [13]. However, the average protein digestibility in these foods was 66.6%, which is lower than average canine protein digestibility [5]. Soybean meal had a lower digestibility than poultry meal while being similar to poultry by-product meal and beef and bone meal in the dog [14]. There was no difference (both were 84%) in the protein digestibility of micronized whole soybeans and corn gluten meal in the cat [15]. Protein utilization was not different when corn gluten meal was compared to fish meal [16] and chicken meal in cats [17]. However, meat meal had superior protein utilization when compared to corn gluten meal in cat food [18].

Although not included in this analysis because of grouping, age does influence protein digestibility. An increased protein digestibility was reported in dogs as they matured to adults (11 to 60 weeks of age) with the high animal based food averaging 84.3% protein digestibility in the dogs at 60 weeks of age [19]. When young adult (4 years of age) dogs were compared to older (13 years of age) there was no difference between the age groups on protein digestibility [20]. In adult cats fed a high energy density food, there was no change in protein digestibility with age. However, when fed a lower density food (the lower energy density was achieved through reduced fat and protein, with increased starch and fiber) the protein digestibility was highest in cats that were 6.3 years of age, reduced in the group averaging 13.3 years of age, and intermediate in the young (average 1.3 years of age) cats. Over all ages and energy densities, the high animal protein foods had protein digestibility of 81.8% [21]. Others have reported a decline in macronutrient digestibility in cats as they aged, which was most significant in fat and energy [22,23].

Interestingly, it is the cat that showed a positive digestibility response to increasing dietary corn gluten meal while digestibility in the dog was unaffected. The cat has an increased ability to digest protein in general, which may influence its capacity to digest corn gluten meal as compared to the dog. In the cat, when manufactured and extruded as in these foods, it appears that after adjusting for metabolic fecal nitrogen, corn gluten meal is nearly 100% digested. In the dog and cat, rice protein concentrate, corn gluten meal, soybean meal, and soybean protein isolate did not negatively influence protein digestibility as compared to animal proteins.

Protein digestibility has been repeatedly shown to be influenced by dietary fiber. In humans, the effect of fiber on protein digestibility was summarized by Gallaher and Schneeman [24] who stated “*it is clear that dietary fiber and fiber-rich foods reduce protein digestibility, often in an approximately linear fashion*.” In pets, fiber has been shown to have a variable effect on protein digestion. Changing the source (beet pulp and corn fibers) and concentration (total dietary fiber 8.4% to 10.2%) did not influence protein digestion in the dog [25]. Increasing dietary cellulose was associated with reduced organic matter digestibility in pets [26]. Cellulose was shown to reduce protein digestion in low energy dense dog food but not in foods with high energy density [27].

Highly fermentable fiber from apple pomace has been shown to significantly reduce protein digestibility [28]. Providing fermentable carbohydrate as oligosaccharides can also reduce protein digestibility [29]. A similar carbohydrate delivery into the large intestine may be accomplished with dietary resistant starch. Resistant starch may be destroyed by extrusion processing but also due to retrograde formation may be created [30]. The carbohydrates in whole grain can influence lower gut microbial metabolism, both as fiber and resistant starch [31]. These fermentable carbohydrates may influence protein digestibility through their lower tract metabolism by the microbiome, which can both trap nitrogen as bacterial protein or liberate nitrogen as ammonia. The grains evaluated here (corn and rice) provide both dietary fiber and resistant starch [32]. Resistant starch may be one of the reasons that Kienzle et al. [27] found an interaction between fiber and dietary carbohydrate on protein digestibility in the dog. This could have been the result of microbial changes associated with the non-absorbed carbohydrate influencing the microbiota, together with the dietary fiber, in a way that increased nitrogen retention through incorporation into bacterial protein in the feces of the dog. Although resistant starch influence on protein digestion in the cat has not been reported, fermentable fiber from plants has a significant effect on both lower gut microbiota and protein digestibility [33]. In that study, it was observed that pectin increased butyrate (generally seen as beneficial) while decreasing protein digestibility in comparison to cellulose. Pectin also increased the fecal microbial genera *Clostridium perfringens, Escherichia coli,* and *Lactobacillus* spp. in comparison to cellulose. This shows that there is at times an unavoidable union between the benefits of lower gut fermentation in producing beneficial postbiotics with the simultaneously reduced protein digestibility. Through the incorporation of whole grains, companion animal foods provide, in addition to proteins and the less fermentable plant fibers, significant sources of highly fermentable fiber, resistant starch, and a number of polyphenols available for microbial fermentation. This fermentation results in the release of postbiotics, which has been defined as the soluble products or by-products of microbial fermentation secreted by bacteria or released after lysis [34]. These postbiotics and their influence on host metabolism and health are an active area of research, which significantly influence the health of the host [33,34,35]. It is possible that the differential effect of these grains on protein digestibility is through the species specific response to their fibers and resistant starch. The respective influence of fermentation of resistant starch from the grains and its interaction with increasing fiber concentration from the grain inclusion is a foundation for ongoing research. The different results for the influence of fiber on digestibility in pets was summarized by de Godoy [36] stating that the different responses are likely the result of fiber levels, type (amount of fermentability), and the dietary matrix.

A significant observation from these data is that the foods tested here had protein digestibility that was within the normal digestibility described by FEDIAF [2]. The species difference response to the increased concentration of whole rice (rice being positive in the dog, negative in the cat) and whole grain corn (no effect in the cat, negative relationship in the dog) did not change protein absorption in a way that in these foods would be expected to be detrimental to the amino acid supply available to the pet. The most significant effect of changing protein digestibility may be providing excess nitrogen for metabolism in the lower gut from the lower digestible foods. If protein is present, but is not absorbed, the dietary amino acids in that protein are not available for the host and provide nitrogen substrate for proteolytic bacteria, which may result in reduced stool quality and increased postbiotics associated with lower tract proteolysis and purification [5,35]. Because nitrogen available for fermentation is a function of protein digestibility and dietary protein concentration, this function of the observed changes in protein digestibility, will change with protein concentration. Excessive lower gut nitrogen may negatively influence pet health through increased concentrations of postbiotic toxins associated with nitrogen metabolism [37]. Moreover, it is likely that other factors, such as the specific microbes supported by the fermentable carbohydrates, influences the level of production of beneficial and toxic postbiotics. Therefore, it is not possible to describe the proteolytic state or “healthiness” of a food based on only a knowledge of protein concentration and protein digestibility. For example, an increased protein concentration from raw meat was concluded to “promote a more balanced growth of bacterial communities and a positive change in the readouts of healthy gut functions” as compared to a food with lower protein and no raw meat [38]. However, these conclusions were based first on an increase in diversity and evenness. Although some disease states (such as inflammatory bowel disease) have a reduction in these indices, it is important to note that in a healthy animal, an increased diversity and evenness could be achieved by holding the healthy microbiota constant, and bringing in a number of pathogenic species to the average relative abundance of those species present. This would increase diversity and evenness while greatly reducing health. The “healthy gut functions” were concluded from an improvement in stool score and a change in the fecal short chain fatty acids. The difference in stool scores seems to be more of a low quality stool in the non-raw food fed dogs, which produced an average score outside of the optimal range rather than an improvement caused by increasing dietary protein. The increased short chain fatty acid lactate observed was associated with changes in the genus *Megamonas*. As this genus is known to respond to available carbohydrate, it is unclear that the shift to a raw meat diet is alone responsible for the observed changes. Although there was a significant increase in protein concentration in the raw food fed dogs, because protein digestibility was not reported, it is not possible to conclude what influence protein present for microbial fermentation had on the measured analytes.

## 5. Conclusions

When prepared in the distribution chain of these ingredients, and manufactured through the extrusion parameters of these foods, plant proteins are similar to animal proteins in protein digestibility. Cats had an increased protein digestibility in response to increasing plant protein, while protein digestibility was not influenced in dogs. As plant protein inclusion was not associated with reduced protein digestibility, it provides a satisfactory source for the complementation of animal protein ingredients in meeting the amino acid needs of pets.

## Figures and Tables

**Table 1:** Pet age and weight.

Pet Descriptor	Canine		Feline	
	Age (years)	Weight (kg)	Age (years)	Weight (kg)
Number of pets	226	226	296	296
Mean	6.4	11.6	7.3	5.4
Minimum	1.1	3.2	1.7	2.9
Maximum	12.8	22.6	12.1	10.1

**Table 2:** Pet food chemical composition (means and standard deviations).

Pet Food Attribute	Canine				Feline			
	Dry (n = 320)		Wet (n = 139)		Dry (n = 256)		Wet (n = 171)	
	Mean	SD	Mean	SD	Mean	SD	Mean	SD
Moisture, %	8.6	1.0	77.6	3.8	6.5	1.2	78.4	2.6
Protein, %	22.1	4.5	5.2	1.2	33.7	4.3	8.2	1.6
Fat, %	14.2	3.4	3.8	1.7	17.9	3.8	4.8	1.4
Ash, %	5.2	1.0	1.2	0.2	5.4	1.7	1.3	0.3
Crude Fiber,%	4.0	3.6	1.1	0.9	3.1	2.7	1.1	0.8
Gross Energy, kcal/kg	4663	209.7	1136	221.2	5115	258.7	1211	168.0

**Table 3:** Protein digestibility coefficients, intakes and fecal weights of canine and feline studies.

All Diets	Canine				Feline			
	Dry (n = 320)		Wet (n = 139)		Dry (n = 256)		Wet (n = 171)	
	Mean	SD	Mean	SD	Mean	SD	Mean	SD
Protein Digestibility	89.8	3.7	86.7	5.0	95.4	3.7	94.8	3.0
Daily Intake, g	197.8	59.5	761.5	249.1	61.2	16.2	217.2	73.2
Daily Fecal Weight, g	103.2	66.2	117.4	67.8	26.6	24.1	26.9	23.4

**Table 4:** The effect of percent of total protein as plant protein and fiber (percent of dry matter) on protein digestibility in the dog.

Statistical Attribute	Canine							
	Dry (n = 320)				Wet (n = 139)			
	Intercept	Crude Fiber	Plant Protein	Plant Protein × Crude Fiber	Intercept	Crude Fiber	Plant Protein	Plant Protein × Crude Fiber
coefficient	90.9	0.015	−0.001	−0.005	88.4	0.142	−0.079	-0.004
std error	1.017	0.231	0.02	0.004	1.342	0.222	0.049	0.006
T ^τ^	89.35	0.067	−0.056	−1.299	65.88	0.642	−1.591	-0.706
*p* >|t|	<0.001	0.947	0.955	0.195	<0.001	0.522	0.114	0.481

^τ^ Student’s t value.

**Table 5:** The effect of percent of total protein as plant protein and fiber (percent of dry matter) on protein digestibility in the cat.

Statistical Attribute	Feline							
	Dry (n = 256)				Wet (n = 171)			
	Intercept	Crude Fiber	Plant Protein	Plant Protein × Crude Fiber	Intercept	Crude Fiber	Plant Protein	Plant Protein × Crude Fiber
coefficient	93.5	−1.222	0.067	0.014	95.4	−0.269	0.042	−0.001
std error	1.087	0.324	0.019	0.005	0.713	0.122	0.032	0.006
T ^τ^	86.0	−3.768	3.474	2.625	133.7	−2.205	1.344	−0.094
*p* >|t|	<0.001	<0.001	0.001	0.009	<0.001	0.029	0.181	0.925

^τ^ Student’s t value.

**Table 6:** Relationship between true protein digestibility and the amount of the dietary protein contributed by whole grains or protein fractions (expressed as dry matter) in canine foods.

Canine	Rice				Corn				Soy			
	Whole Rice (n = 301)		Rice Protein Concentrate (n = 7)		Whole Corn (n = 219)		Corn Gluten Meal (n = 178)		Soybean Meal (n = 85)		Soybean Protein Isolate (n = 11)	
	Intercept	Slope	Intercept	Slope	Intercept	Slope	Intercept	Slope	Intercept	Slope	Intercept	Slope
coefficient	88.5	0.105	92.3	−0.030	89.0	−0.085	89.5	−0.029	87.5	−0.038	91.1	0.058
std error	0.316	0.036	3.07	0.11	0.451	0.036	0.804	0.03	0.716	0.048	1.19	0.05
T^τ^	280.4	2.9	30.0	−0.27	197.3	−2.367	111.4	−0.948	122.1	−0.785	76.5	1.157
*p* > |t|	<0.001	0.003	<0.001	0.798	<0.001	0.019	<0.001	0.345	<0.001	0.435	<0.001	0.277

^τ^ Student’s t value.

**Table 7:** Relationship between true protein digestibility and the amount of the dietary protein contributed by whole grains or protein fractions (expressed as dry matter) in feline foods.

Feline	Rice				Corn				Soy			
	Whole Rice (n =341)		Rice Protein Concentrate (n = 12)		Whole Corn (n = 107)		Corn Gluten Meal (n = 242)		Soybean Meal (n = 23)		Soybean Protein Isolate (n = 19)	
	Intercept	Slope	Intercept	Slope	Intercept	Slope	Intercept	Slope	Intercept	Slope	Intercept	Slope
coefficient	95.7	−0.16	96.4	−0.043	94.6	0.028	92.2	0.093	93.7	−0.014	95.5	0.069
std error	0.283	0.068	2.576	0.066	0.666	0.2	0.52	0.014	1.294	0.25	0.979	0.048
T ^τ^	337.8	−2.3	37.4	−0.646	142.1	0.139	177.3	6.471	72.4	−0.056	97.6	1.433
*p* >|t|	<0.001	0.02	<0.001	0.533	<0.001	0.89	<0.001	<0.001	<0.001	0.956	<0.001	0.17

^τ^ Student’s t value.
